# Acute and Chronic Effects of Betel Quid Chewing on Brain Functional Connectivity

**DOI:** 10.3389/fpsyt.2020.00198

**Published:** 2020-03-17

**Authors:** Adellah Sariah, Shuixia Guo, Jing Zuo, Weidan Pu, Haihong Liu, Edmund T. Rolls, Zhimin Xue, Zhening Liu, Xiaojun Huang

**Affiliations:** ^1^Mental Health Institute of the Second Xiangya Hospital, Central South University, Changsha, China; ^2^Department of Mental Health and Psychiatric Nursing, Hubert Kairuki Memorial University, Dar es Salaam, Tanzania; ^3^MOE-LCSM, School of Mathematics and Statistics, Hunan Normal University, Changsha, China; ^4^Key Laboratory of Applied Statistics and Data Science, Hunan Normal University, Changsha, China; ^5^Department of Psychiatry, Brain Hospital of Hunan Province, Changsha, China; ^6^Medical Psychological Institute, Second Xiangya Hospital, Central South University, Changsha, China; ^7^Mental Health Center of Xiangya Hospital, Central South University, Changsha, China; ^8^Oxford Centre for Computational Neuroscience, Oxford, England; ^9^Department of Computer Science, University of Warwick, Coventry, England

**Keywords:** functional brain imaging, basal ganglia, orbitofrontal cortex, betel quid, arecoline, resting-state fMRI

## Abstract

**Background:**

The active alkaloid in Betel quid is arecoline. Consumption of betel quid is associated with both acute effects and longer-term addictive effects. Despite growing evidence that betel quid use is linked with altered brain function and connectivity, the neurobiology of this psychoactive substance in initial acute chewing, and long-term dependence, is not clear.

**Methods:**

In this observational study, functional magnetic resonance imaging in a resting-state was performed in 24 male betel quid-dependent chewers and 28 male controls prior to and promptly after betel quid chewing. Network-based statistics were employed to determine significant differences in functional connectivity between brain networks for both acute effects and in long-term betel users versus controls. A support vector machine was employed for pattern classification analysis.

**Results:**

Before chewing betel quid, higher functional connectivity in betel quid-dependent chewers than in controls was found between the temporal, parietal and frontal brain regions (right medial orbitofrontal cortex, right lateral orbital frontal cortex, right angular gyrus, bilateral inferior temporal gyrus, superior parietal gyrus, and right medial superior frontal gyrus). In controls, the effect of betel quid chewing was significantly increased functional connectivity between the subcortical regions (caudate, putamen, pallidum, and thalamus), and the visual cortex (superior occipital gyrus and right middle occipital gyrus).

**Conclusion:**

These findings show that individuals who chronically use betel quid have higher functional connectivity than controls of the orbitofrontal cortex, and inferior temporal and angular gyri. Acute effects of betel quid are to increase the functional connectivity of some visual cortical areas (which may relate to the acute symptoms) and the basal ganglia and thalamus.

## Introduction

Betel quid (BQ) is a psychotropic substance, extensively consumed by more than 600 million people worldwide (1). Right after consumption, users of BQ have reported experiencing decreased thinking ability, disturbed mental processes, increased vigilance, body relaxation, enhanced motor responses, and a boosted sense of wellness (2). Substance dependence features including tolerance, craving, and drug-seeking behaviors as well as withdrawal symptoms have been acknowledged by habitual users of BQ (3). Many psychoactive substances act on the brain's reward pathway during acute administration, an effect that may be different in habitual users (4). The basal ganglia, extended amygdala, and the prefrontal cortex have been implicated in the initial stages, development, and habitual use of addictive substances (5). During the initial stages, the individual engages in voluntary substance use behaviors (6). Such behaviors may be accompanied by intense feelings which once experienced, may enhance recurrent substance use (7). Arecoline is the principal active component in BQ (8). It facilitates the release of dopamine (DA) (9) by binding to M_5_ muscarinic acetylcholine receptors on GABA terminals on DA neurons in the ventral tegmental area (VTA) (10). DA concentration is increased in the VTA and other projection areas through a series of mechanisms carried out by the mesocorticolimbic system [VTA, nucleus accumbens (NAc), and prefrontal cortex (PFC)], which is considered to be a principal pathway of drug reward (11). Additionally, cholinergic and inhibitory GABA'ergic inputs greatly regulate the mesolimbic dopaminergic neurons (12), which are known for their important role in processing rewards, reinforcement learning, (13) and dependence (14). Moreover, acute administration of psychoactive drugs has been found to activate brain areas connected to the mesocorticolimbic neural networks, implicated in drug rewards (15). Therefore, the need to use psychoactive substances repetitively and the compulsivity that is demonstrated in individuals addicted to drugs may be elucidated by the involvement of the reward and habit pathways in the brain (5). In contrast to the increased dopaminergic transmission in the NAc during acute exposure to drugs, chronic drug use is linked with less rewarding effects which result from reduced DA levels (16, 17). Chronic drug use is known to diminish the ability of the brain to control drug use behaviors, leading to increased risk for compulsive behavior that characterizes addiction (6). At first, it was believed that losing control over drug use stemmed from impairment in the subcortical reward brain region. However, findings from addiction studies have demonstrated the crucial role of the PFC in modulating the limbic reward regions and executive functions. Disruption of the PFC has been associated with loss of inhibitory control observed in drug-addicted individuals who have relapsed (18).

Resting-state functional connectivity (FC) studies have found that the majority of addictive drugs lead to reward, emotional and cognitive dysregulation (19). The mesocorticolimbic (MCL) system has been implicated to play an important role in drug addiction. The interaction among and between the MCL regions and other subcortical and cortical structures that manifests as circuit-level FC alterations have been observed in the reward circuit of drug addicts (19). The principal reward network is connected to other reward brain areas encompassing the subgenual anterior cingulate cortex (ACC), medial orbitofrontal cortex (mOFC), and medial PFC (mPFC) (20). For instance, increased resting-state FC was observed between NAc and the ventral mPFC (vmPFC) (rostral ACC and mPFC) of heroin addicts (21). Likewise, abstinent cocaine-users displayed greater resting-state FC between the ventral striatum and the vmPFC (22). Apart from increased striatal-PFC FC, a study investigating FC in prescription opioid users found reduced FC between NAc and the subcortical (hippocampus and amygdala) and cortical (cingulate, parietal, prefrontal) regions (23).

Drugs of addiction are also characterized by emotional dysregulation emanating from altered FC between the amygdala and PFC regions (19). Interaction of the amygdala with mPFC, hippocampus, cingulate, and insula regions has been linked with emotional processing and regulation, and generation of affective states (24). The hippocampus (involved in memory and learning) and the dorsal ACC (involved in cognitive control) are thought to be impaired in addiction, where a greater saliency value of drugs accompanied by a weaker inhibitory control leads to compulsive drug-seeking behavior (25, 26). The amygdala and its connections are fundamental elements perpetuating drug use, and previous studies propose that aberrant amygdala-mPFC FC may play a crucial role in emotional dysregulation frequently observed in drug addicts (19). Reduced FC strength was reported between the amygdala and regions of mPFC (including vmPFC and rostral ACC) in individuals addicted to cocaine (27), and heroin abusers (28). Similarly, extensive reduction of FC was displayed between the amygdala and several regions, including ventrolateral, medial, and dorsolateral PFC (dlPFC) regions in individuals addicted to prescription-opioid (23). In this study, longer periods of opioid use were linked with greater amygdala-vmPFC (specifically the subgenual ACC) FC reductions.

Apart from reward and emotional deficits, individuals addicted to psychoactive drugs are known to display neural dysfunction linked with cognitive control (29). The cognitive control network includes the ACC, lateral PFC, and parietal areas (19). For instance, decreased resting-state FC was observed between the ACC and dlPFC of heroin users relative to controls (21). Moreover, substantially decreased FC was observed within and between lateral PFC and parietal regions, such that decreased interhemispheric connectivity between lateral PFC areas was associated with a greater frequency of self-reported cognitive deficits (30) in cocaine addicts. A similar characteristic was displayed in abstinent heroin users such that FC was reduced between the lateral PFC and parietal regions. The observed reduction in FC matched a reduction in gray matter density in the same regions, with a longer duration of use predicting a greater reduction in both parameters (31).

A number of psychoactive substances have been linked with FC alterations in addicts. Specifically, compared to healthy controls, cocaine users displayed decreased FC within corticostriatal reward circuitry (27, 32), which has been associated with compulsive use of drugs and relapse (32). Apart from the reward circuitry, users of cocaine demonstrated altered FC between vital regions in the salience network and cortical regions (involved in decision making) (33); and within cortical brain areas involved with executive control (such as the cognitive control and attentional salience networks) (30, 33). Similarly, cocaine dependence has been associated with disruption among the default mode, salience and emotional networks where cocaine-dependent individuals displayed decreased connectivity between rostral ACC and salience network; posterior cingulate cortex (PCC) and executive control network (ECN); and bilateral insula and default mode network (DMN) (34). Moreover, compared to controls, the ventral striatum of individuals with cocaine dependence exhibited reduced FC with the hippocampal, parahippocampal gyrus, vmPFC, and increased FC with the visual cortex (35). In alcohol dependence, individuals are often characterized by an impulsive drive to consume alcohol and a lack of self-control towards its consumption despite negative consequences (36). Evidence shows that individuals with alcohol dependence showed increased within-network FC in the salience network (SN) (including insula, hippocampus, and temporal lobe); anterior DMN (including superior frontal gyrus (SFG), ACC, medial frontal gyrus (MFG), and superior medial gyrus); posterior DMN (involving middle cingulate cortex, PCC, precuneus, insula, caudate, superior temporal gyrus (STG), and thalamus); orbitofrontal cortex (OFCN) (including middle and superior orbital gyrus, insula, amygdala); amygdala-striatum (ASN) (including putamen, amygdala, caudate, hippocampus, and inferior temporal gyrus (IFG); and left executive control (LECN) networks (consisting of the angular gyrus) (36). Relative to controls, cannabis abusers demonstrated increased resting-state FC of subcortical regions. Specifically, the cannabis abusers displayed greater local functional connectivity density (lFCD) than controls in the ventral striatum (NAc location), midbrain (SN/VTA location), brainstem, and thalamus (37). Results from a study investigating FC of the DMN revealed increased FC in the right hippocampus, while reduced FC was found in the right dorsal ACC and left caudate of chronic heroin users relative to controls (21). Increased FC was observed between dorsal ACC (dACC)-right anterior insula (AI), the dACC-thalamus, the dACC-left AI, and the right AI-left AI of nicotine addicts. Increased FC was associated with risky decision making (38).

The effect of BQ use on brain functional connectivity during acute administration is not well understood. Studies investigating the acute effects of BQ have largely focused on the frontal and default mode networks, giving subcortical regions less attention. For instance, based on previous findings from independent component analysis (ICA), acute use of BQ among naïve chewers was associated with increased and decreased FC in the frontal and default mode networks respectively (39). Evidence from addiction studies has linked initial drug use with activation of the reward pathway which primarily involves the interaction between subcortical and frontal cortical structures (6). Additionally, resting-state fMRI studies investigating chronic effects of BQ in the brain have yielded inconsistent results. For instance, compared to controls, individuals with betel quid dependence (BQD) had decreased FC in the DMN (40, 41), parietal network (42), and between the anterior cingulate cortex (ACC) and DMN (43); while increased connectivity was mostly displayed in networks including the visual (41), frontoparietal, occipital/parietal, frontotemporal, temporal/limbic, and frontotemporal/cerebellum (42), and between the ACC and regions of the reward network (41). The different FC results may have been influenced by differences in subjects across studies (sample size, age, gender, variations in BQ preparation, dependence level, duration of BQ exposure and the use of other substances, e.g., alcohol and tobacco) and use of different analysis methods, such as ICA (39, 40, 42), functional connectivity density mapping (44), graph theoretical analysis (GTA), and network-based statistics (NBS) (41).

Persistent psychoactive substance use has been linked with impaired brain function which disrupts the ability to wield self-control over drug use behaviors that typifies addiction (6).

Previous neuroimaging studies have investigated separately the acute and chronic effects of BQ on brain functional connectivity. Specifically, acute and chronic effects of BQ were mostly explored in the DMN and different parts of the brain respectively. This is the first study to examine both the acute and chronic effects of BQ concurrently. We explored the whole brain rather than predefined systems, with the aim of elucidating the impact of both initial and chronic BQ use on brain FC. Specifically, our first aim was to examine functional connectivity during initial BQ use among naïve chewers. Second, we aimed to explore the differences in FC between the naïve and BQ dependent chewers. We used NBS to identify FC differences (45). The results may provide further evidence and understanding of the neural mechanisms involved during initial BQ chewing and BQD.

## Materials and Methods

### Aim, Design, and Setting of the Study

This is an observational neuroimaging study that aimed to examine the effects of both acute and chronic BQ chewing in the whole brain. Recruitment of participants and data collection was carried out between January 2015 and March 2016 at the Second Xiangya Hospital of Central South University, located in Changsha city, Hunan Province, China.

### Characteristics of Participants

All participants in this study were male. The following criteria for inclusion and exclusion of participants have been described in our previous papers (39, 42). Twenty-five individuals with BQD had to meet the following inclusion criteria: (1) 18–40 years of age; (2) Han Chinese ethnicity; (3) accomplished nine or more years of education; (4) right-hand dominant; (5) diagnosed with BQD as individuals using BQ at least 1 day at a time for more than 3 years and with a score of 5 or higher on the Betel Quid Dependence Scale (BQDS). The BQDS is a 16-item self-administered tool made up of three parts: physical and psychological urgent need, increasing dose and maladaptive use (46). Exclusion criteria included: (1) a history of neurological disorder or other serious physical illness; (2) a history of any mental disorders; (3) a history of substance abuse other than BQ; (4) a contraindication to MRI.

Thirty healthy controls were enrolled from the community in the Changsha City area. The inclusion and exclusion criteria for controls corresponded to those of the BQD group. The only exception was that controls would not have a diagnosis of BQD or have a family history of psychiatric illness amongst their first-degree relatives. All study participants were asked not to use any psychoactive substance during the 24-h period prior to scanning.

BQ can induce some physiological and psychological changes to users. But a half fruit of BQ is unlikely to induce severe adverse effects in healthy young men, even if used for the first time. We recorded the participants' heart rates and blood pressures just before the first scan and right after the second scan (about 30 min after using the betel quid) so as to monitor and rule out any physiological changes to users. Statistical analysis showed that there were no significant differences between the first and second measures of heart rate or blood pressure (**Table 1** below). It has been reported that “The onset (of physiological effect) was within 2 min after chewing, peak effect was reached within 4–6 min and the effect lasted for an average of 16.8 min (47).” The absence of changes in heart rate or blood pressure may have resulted from the long interval (about 30 min) between the betel quid chewing and the recording. We also administered behavior questionnaires including the Beck Depression Inventory and Beck Anxiety Inventory before betel quid chewing to assess the participants' emotional status.

**Table 1:** Demographics and clinical characteristic of participants.

	BQD (Mean ± SD)	HC (Mean ± SD)	*t*/χ^2^	*P-*value
Age (years)	23.50 (3.88)	24.93 (2.60)	−1.58^a^	0.12
Gender (male/female)	24/0	28/0		
Education (years)	15.13 (1.73)	16.26 (1.32)	−2.66^a^	0.01*
Betel Quid Dependence Scale	7.58 (2.17)	N/A		
Duration of Betel Quid (years)	7.13 (3.79)	N/A		
Beck Depression Inventory	10.38 (6.75)	3.75 (4.60)	4.20^a^	0.00*
Beck Anxiety Inventory	28.588(6.25)	23.11 (2.64)	4.45^a^	0.00*

SD, standard deviations; N/A, not applicable.

a Independent-samples t-test.

*P < 0.05.

This study was conducted in accordance with recommendations of the Helsinki Declaration established in 1964, and its later amendments or comparable ethical standards. Approval to conduct this study was obtained from the Ethics Committee of the Second Xiangya Hospital of Central South University. Before inclusion in the study, written informed consent was provided by each participant.

### Image Acquisition and Preprocessing

HC1 and BQD1 were defined as controls and participants with BQD respectively, who were scanned before BQ chewing. HC2 and BQD2 were defined as controls and participants with BQD respectively scanned after BQ chewing. In theory, we can compare any pair of conditions. However, in the main text, we describe the results for HC1 versus HC2 and HC1 versus BQD1. The results for other comparisons are shown in the supplementary materials. The following explanation about image acquisition and preprocessing parameters have also been described in our previous papers (39, 42). Resting-state fMRI scans were carried out for all participants before and after BQ chewing. HC1 and BQD1 were asked to chew the dried BQ along with its husk and swallow the saliva quickly in no more than 3 min. The BQ was an industrially wrapped product that has been described before by (48). Subsequently, the residual BQ was spat out, and 3 min later participants underwent the second fMRI scan which resulted in HC2 and BQD2.

Resting-state images were obtained from a Philips Gyroscan Achieva 3.0-T scanner in the axial direction. The following imaging parameters were used for the gradient-echo echo-planar imaging sequence: matrix size = 64 × 64, repetition time = 2,000 ms, echo time = 30 ms, flip angle = 90°, gap = 0 mm, field of view = 24 cm × 24 cm, number of slices = 36, and slice thickness = 4 mm. Earplugs and foam pads were utilized to lessen scanner noise and head motion respectively. Participants were asked to lie flat on their back motionless with their eyes closed. The maximum time for each resting-state fMRI scan was 500 s, and generally, 250 image volumes were acquired.

The Data Processing Assistant for Resting-State fMRI (DPARSF) toolbox (49) was utilized to preprocess the fMRI imaging data by way of Statistical Parametric Mapping (SPM8) (50). The first 10 images were removed to allow for scanner adjustment and for participants to gain familiarity with the scanner environment. Slice-timing correction and realignment for the head motion were performed to the residual 240 image volumes. The following measures were taken to minimize the effect of head motion on functional connectivity: First, the following criteria had to be met for realignment (1): a maximum displacement in the x, y, or z-axis of less than 2 mm and (2) angular rotation about each axis of less than 2°. Initially, the scan was performed among 25 BQD and 30 HCs, nevertheless, 1 BQD participant and 2 HCs had to be excluded from the study owing to greater than 2° and 2mm of rotations and translations respectively during fMRI scanning. Second, we utilized the Friston 24-parameter model (51) to regress out head motion effects from the realigned data (i.e., 6 head motion parameters, 6 head motion parameters one-time point before, and the 12 corresponding squared items) based on recent reports that higher-order models demonstrate benefits in removing head motion effects (52). Third, the head motion was also controlled at the group-level by using the mean framewise displacement (FD) as a covariate. These measures were strict enough to control artifacts caused by head movements. We compared head motion between the BQD1-2 and HC1-2, which was measured by mean FD derived from Jenkinson's formula (53), and no difference was detected between the groups. A scrubbing procedure was performed, where we calculated DVARS (a temporal derivative of time courses and variance across voxels) (54) to measure the rate of change of the BOLD signal across the entire brain for each frame of data. This revealed only a very small proportion of our data had movement contamination. When we compared the results obtained from the original data and the movement scrubbed data, there were no notable differences. Thus, all the results are obtained from the original data in this paper.

The data were spatially normalized into standard coordinates using the Montreal Neurological Institute echo-planar imaging template in the SPM package and was then resampled into 3 mm × 3 mm × 3 mm voxels. The preprocessed images were smoothed using a 4mm Gaussian kernel before the statistics. Subsequently, the BOLD signal of each voxel was first detrended to eliminate any linear trend. These signals were then passed through a band-pass filter of 0.01–0.08 Hz to decrease low-frequency drift and high-frequency physiological noise. Lastly, nuisance covariates (Friston 24-head motion parameters, white matter and cerebrospinal signals) were regressed out from the BOLD signals.

### Whole-Brain Functional Network Construction

The revised automated anatomical labeling atlas (AAL2) (55) was used to parcellate the brain into 94 regions of interest (ROI) (47 in each hemisphere). The mean time courses were obtained from each ROI by extracting the signal average of all voxels within the region. The AAL2 atlas provides an upgraded parcellation of the orbitofrontal cortex for the automated anatomical labeling atlas (56). The new parcellation of the orbitofrontal cortex is based on anatomical evidence (57). The anatomical regions defined in each hemisphere and their labels in the AAL2 are provided in the supplementary materials (**Table S4**).

### Acute Impact of BQ

The acute impact of BQ was estimated by comparing HC1 versus HC2. Pearson correlation coefficients were calculated between all pairs of ROIs, to acquire 94 × 94 correlation matrices *r_ij_*,*i*, *j* = 1, 2….,94, indicating the FC strength for each pair (connectivity between any two brain regions) of regions for each participant. Then, the FC with significant differences (p-value of less than 0.05) before and after chewing BQ were selected by performing a paired t-test and the difference network was constructed. The number of all FCs for each node in the different network was defined as the degree of this brain region.

### Differences of Functional Connectivity in Chronic BQ Users and Controls

These differences were estimated by comparing the difference between HC1 vs BQD1. The 94 × 94 Pearson correlation coefficients were initially calculated between all pairs of ROIs. Then, FCs with significant differences (p-value of less than 0.05) between HC1 and BQD1 were selected by performing a two-sample t-test and the difference network was constructed. The number of all FCs for each node in the difference network was defined as the degree of this brain region.

### Network-Based Statistics

At present, studies using neuroimaging data to construct functional or structural networks are many, and most of them are aimed at finding different connections between the two groups of networks. When we test each connection in the graph of the network at the same time, the family-wise error rate is generated. The network-based statistic is an effective way to control the family-wise error rate, depending on the degree of association between the connections of interest (45). The specific steps are as follows. First, a Fisher's r to z transform for each connection in the network was performed, and a t-test was performed for the differences between the two groups for each connection. The test statistic computed for each pairwise association was then thresholded to formulate a set of suprathreshold links. Components present in the set of suprathreshold links were ascertained using a breadth-first search, and the number of links they comprise (or size) was stored. Thereafter, permutation testing was used to assign a p-value controlled for the FWE to each connected component based on its size. A total of M random permutations was created independently, where for each permutation, a random exchange was done for the group to which every participant belongs. The required test statistic for each permutation was recalculated, then the same threshold was applied to define a set of suprathreshold links. The maximal component size in the set of suprathreshold links extracted from each of the M permutations was ascertained and stored, thereby earning an empirical estimate of the null distribution of maximal component size. Lastly, to estimate the p-value of an observed component of size k, the total number of permutations was detected showing a greater maximal component size than k and normalizing by M. Networks with significant inter-group differences were detected if the p-value was smaller than the given 0.05 threshold.

### Support Vector Machine (SVM) Classifier

In order to study how much difference there is between the different groups, we employed a widely used SVM classifier. SVM is a learning machine for two-class problems for pattern classification analysis.

We used an SVM toolkit called libsvm composed by Chih-Jen Lin from Taiwan University (58) (http://www.csie.ntu.edu.tw/~cjlin/libsvm/). Specifically, the whole brain FC was applied to the raw input matrix. Features that appeared statistically significant (a smaller p-value than the threshold for a two-sample t-test) were picked. Various types of kernel (linear, t=0; polynomial, t=1; radial basis function, t=2) and different trade-off parameter C (0.001, 0.01, 0.1, 1, 10, 100, 1,000, 10,000) were tried to attain the highest accuracy rate. A leave-one-subject-out cross-validation technique was applied to ascertain how the test performs as well as to validate the classifier, where the classifier was trained on all subjects except one, who was then used for the test data. The mean discrimination accuracy, sensitivity, specificity and AUC (area under ROC curve) were obtained for the entire sample.

Selecting the generalization rate as the statistic, permutation tests were employed to estimate the statistical significance of the observed classification accuracy. In the permutation testing, the class labels of the training data were randomly permuted prior to training. Cross-validation was then performed on the permuted training set, and the permutation was repeated 100 times. The *p-*value represents the probability of observing a classification prediction rate in the permutation testing no less than the discrimination accuracy. If the *p-*value is smaller than the significance level, we reject the null hypothesis that the classifier could not learn the relationship between the data and the labels reliably and declare that the classifier learns the relationship with a probability of being wrong of at most *p*.

## Results

The mean age for BQD chewers and HC was 23.5 ± 3.88 years and 24.9 ± 2.60 years respectively. Individuals with BQD displayed a mean BQDS score of 7.58 ± 2.17 and a mean duration of BQ use of 7.13 ± 3.79 years (**Table 1**).

### Acute Impact of BQ

**Figure 1:**
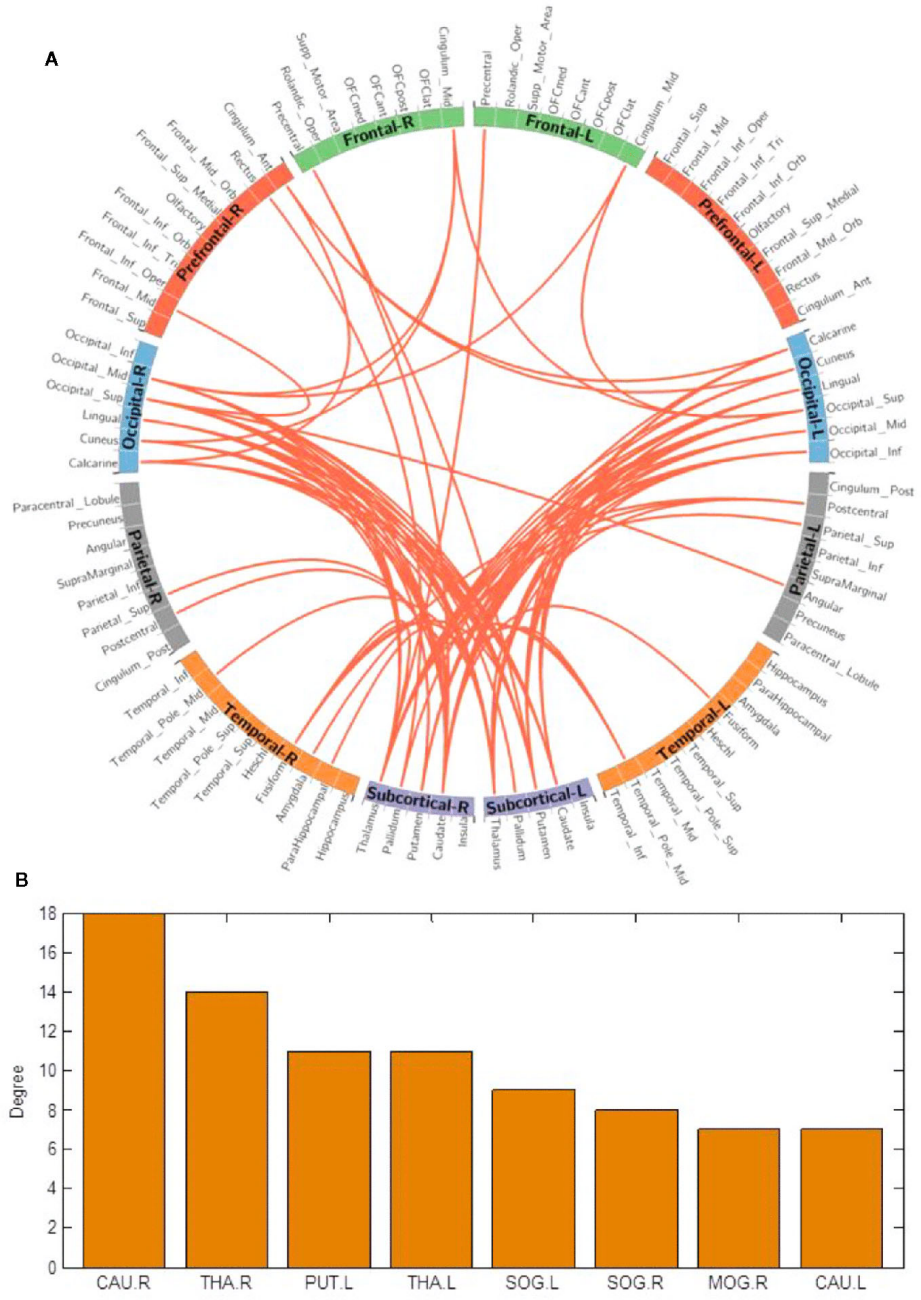
**(A)** Network-based functional connectivity differences after acute administration of betel quid to naive participants. Orange indicates an increase in functional connectivity which was found between the subcortical and occipital brain regions. **(B)** The degree of different areas for acute use (ROIs with the highest degree). CAU, caudate; L/R, left/right; MOG, middle occipital gyrus; PUT, putamen; SOG, superior occipital gyrus; THA, thalamus.

**Figure 2:**
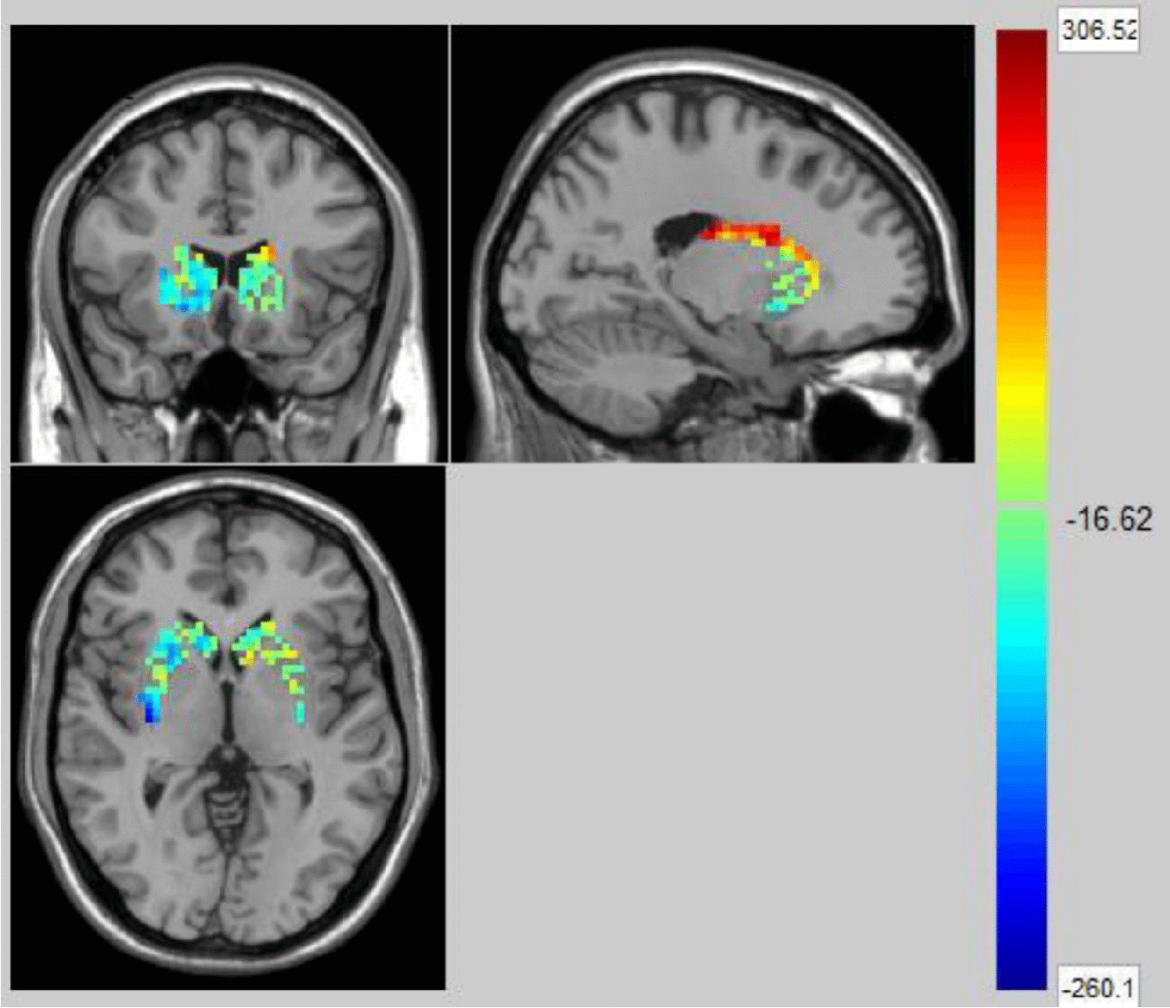
The t map (link-wise FDR corrected, q=0.05) in Healthy Controls before and after acute betel quid chewing. A significant difference was detected with connectivity between the caudate and putamen. The colorbar represents the t value for each region of interest (ROI).

We used the NBS method to assess specific network connections for acute impact. Compared with HC1, HC2 displayed higher functional connectivity strength between subcortical regions including the basal ganglia and thalamus, and occipital brain regions, after chewing BQ as shown in **Figure 1A**. The brain regions comprised of 37 nodes and 55 connections that included many connections involving the basal ganglia (corrected p value < 0.001). The ROIs with the highest degree are shown in **Figure 1B** including bilateral caudate (CAU), thalamus (THA), left putamen (PUT), bilateral superior occipital gyrus (SOG) and middle occipital gyrus (MOG). Because the right caudate has the biggest degree in **Figure 1**, it was used as an example to calculate the connectivity between the right caudate and the other voxels in the whole brain. We performed this analysis to show whether the voxel-wise analysis is consistent with the NBS analysis. We used a pairwise t-test to compare the difference between HC1 and HC2. The t map (link-wise FDR corrected, q=0.05) is shown in **Figure 2**. A significant difference was detected with connectivity involving the caudate and putamen. No association was found between the baseline scores and FCs.

### Differences of Functional Connectivity in Chronic BQ Users and Controls

**Figure 3:**
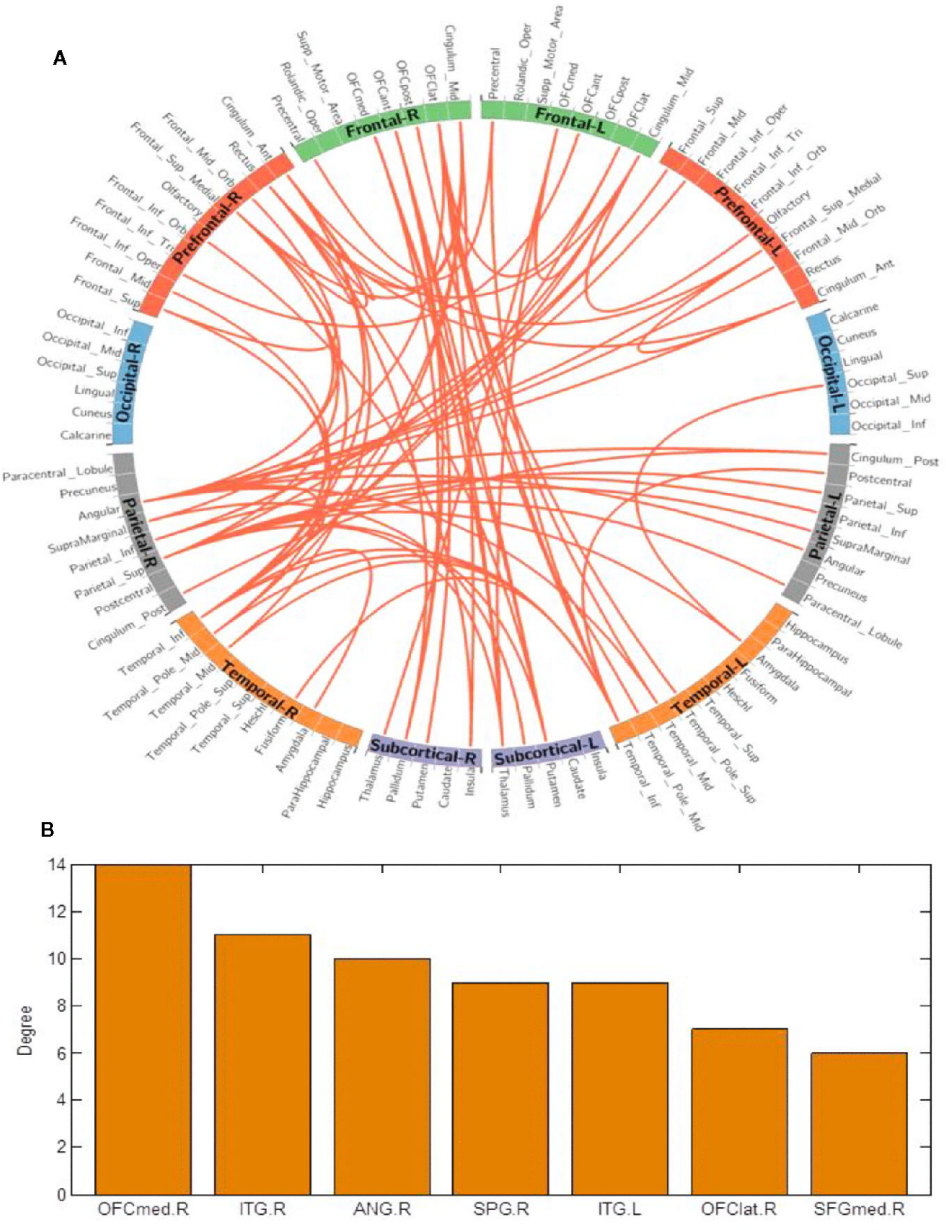
**(A)** Network-based connection differences for chronic betel quid users minus controls. Orange indicates an increase in functional connectivity which was found between the frontal, parietal and temporal brain regions. **(B)** The degree of different areas for chronic use [regions of interest (ROIs) with the highest degree] The right OFCmed is the combination of the sum degree of rectus, OFCmed, OFCant, and OFCpost). ANG, angular gyrus; ant/post, anterior/posterior; ITG, inferior temporal gyrus; L/R, left/right; med/lat, medial/lateral; OFC, orbitofrontal cortex; SFG, superior frontal gyrus; SPG, superior parietal gyrus.

Using the NBS method, higher functional connectivity strength in BQD1 compared with HC1 was found between the temporal, parietal and prefrontal brain regions, as shown in **Figure 3**. The regions comprised of 55 nodes and 79 connections (corrected p-value is 0.035). The ROIs with the highest degree are shown in **Figure 3B** including the right medial OFC, right lateral OFC, angular gyrus, superior parietal gyrus (SPG), SFG, and bilateral ITG. There was no association between the baseline scores and FCs.

Significant links during acute and chronic BQ chewing are provided in supplementary materials 456 (**Figure S1** and **Table S2**).

### Network-Based Classification

Receiver operating characteristic curves (ROC) were charted for network classification analysis, so as to ascertain whether graph-based network metrics might act as biomarkers for discriminating different groups. The ROC analysis was performed for each metric (i.e., one-dimensional characteristic) displaying significant differences between groups. For each particular metric, a range of thresholds was employed to allocate each participant into either the first or the second group. An initial linear discriminant analysis was carried out to yield an overall estimate of group separation. The highest degrees of separability (AUC) were observed between HC1 and HC2 (0.7551), as well as between HC1 and BQD2 (0.8646). HC2 versus BQD2 and BQD1 versus BQD2 portrayed the lowest AUC (**Table S3** and **Figure S2** in supplementary materials).

## Discussion

This study identifies the neurobiological effects involved in the initial acute effects of BQ chewing and in differences in those who are BQ dependent from controls when BQ was not being administered. For the purpose of this study, we only compared HC1 versus HC2, and HC1 versus BQD1, because our interest lies in the acute impact of BQ chewing, and in differences between long-term BQ and non-BQ users. NBS was used because it is a more sensitive approach than FDR.

The results from the SVM showed that the biggest difference between groups was observed between healthy controls who did not chew BQ and individuals who chewed BQ. For instance, HC1 versus BQD2 and HC1 versus HC2 have greater AUC values compared to those who chewed BQ (HC2 versus BQD2 and BQD1 versus BQD2). A small difference was detected between those who chewed BQ. Previous studies have also reported the difference in connectivity between BQ dependent chewers and healthy controls (41–43), as well as between non-chewer healthy controls and healthy controls who chewed BQ (39). This is consistent with the hypotheses that BQ can alter the brain once it is consumed; or with the hypothesis that there are differences between individuals that lead some to consume BQ. AUC has previously been suggested as the preferred measure of diagnostic accuracy in psychiatry and forensic psychology, with reports considering AUC values greater than 0.7 as having strong effects in test performance (59).

### Effects of Acute BQ Chewing on Brain Functional Connectivity

Increased functional connectivity among naive BQ chewers was mostly observed between subcortical regions including the basal ganglia and thalamus (CAU, PUT.L, and THA) and the visual cortex (SOG and MOG.R). This effect of BQ is consistent with previous reports about increased activity in these regions during acute cocaine administration (60). For the majority of psychoactive drugs, the acute effects involve the activation of reward pathways (6). The reward pathways in the brain include the basal ganglia (including the striatum), the limbic system (amygdala) and parts of the PFC (61). The basal ganglia are known for modulating the rewarding effects of drug use and also play a role in habit formation (dorsal striatum) (5). The striatum is involved in reward-related learning, as well as contributing to the development and maintenance of addictive behaviors (62). In particular, the putamen and caudate of naive BQ chewers demonstrated significantly increased FC, portraying the role of the striatum in the reward pathway during acute drug administration (6). DA neurons in the VTA play an important role in processing drug rewards (63), and increased DA in the striatum has been linked with subjective feelings of pleasure, euphoria, or a “high” resulting from drug use (64) and alcohol-associated cues (65). Our results agree with previous studies where activation of the ventral striatum significantly correlated with smoking motivation for pleasurable relaxation (66), supporting the reported psychological effects experienced immediately after BQ chewing (2). Similarly, acute alcohol influences neuronal activity in the ventral striatum which is known to project to regions believed to regulate motor responses, motivation and executive functions (67). Compared to non-users, individuals with substance use disorders displayed reduced functional connectivity between the nucleus accumbens and the frontal cortical regions responsible for controlling cognition (68). Such findings provide evidence that differences in the connections between the reward processing and cognitive-behavioral control areas may play a crucial role in the development of substance use disorders including betel quid dependence.

Compared to the frontal and striatal brain areas, the visual cortex (69) has received attention to a lesser degree in substance use and addiction neuroimaging studies. Our study found significantly increased FC involving the visual cortex of controls immediately after BQ chewing, which is consistent with results by Huang et al. (39) and also backing up the reported heightened alertness experienced by BQ chewers (8). Increased FC in the visual cortex has also been demonstrated after acute alcohol consumption (70). We suggest that BQ may enhance alertness which in turn activates the visual cortex, however, this requires further investigation. Drug cue exposure studies have also documented activation of the visual cortex in substance abusers when presented with visual drug cues as compared to neutral cues (71).

### Differences of Functional Connectivity in Chronic BQ Users and Controls

Our study found significantly higher FC in the networks involving the right medial OFC, right lateral OFC, and right SFG of BQ dependent individuals. Analogous results have been reported by (39). The OFC, whose disruption leads to maladaptive and impulsive decision making (72), is known for its function in signaling the value of expected outcomes or consequences (73), motivational behavior (74), salience attribution (75), emotional regulation, and decision making (together with the amygdala and insula) (61, 76, 77). It has numerous projections to the striatum (75), and each of its sub-regions performs distinct functions; for example, the OFCmed is known for its role in monitoring reward stimuli whereas the lateral OFC evaluates punishing stimuli (78). For instance, individuals carrying out a monetary decision-making task exhibited activation of the OFCmed to positive reward outcome whereas activation of the lateral OFC was observed during monetary loss outcome (79). Addiction studies have also shown that the enhanced expectation value of a drug in the reward (ventral pallidum, NAc, and VTA), motivation (medial OFC, VTA, ventral ACC, dorsal striatum, SN, and motor cortex), and memory (medial OFC, amygdala, dorsal striatum, and hippocampus) circuits overcomes the control circuit (dlPFC, inferior frontal cortex, ACC, and lateral OFC) resulting in compulsive drug use and loss of control (80). Furthermore, compared to controls, individuals with cocaine dependency showed significantly decreased interhemispheric resting-state functional connectivity of the prefrontal cortex and the dorsal attention network encompassing medial premotor and posterior areas, as well as the bilateral frontal (30). Additionally, resting-state studies have portrayed dysfunctional network connectivity across the brain during both acute and chronic nicotine exposure, where the effects mostly appeared to involve the networks linked with attention, cognitive control, ACC, and insula (81). This relates to the enhanced attention that is commonly experienced by smokers (81) and continues to support the notion that substance use is associated with various alterations in connectivity between significant regions of the brain. The brain reward system is not only activated by the drugs, but also stimuli associated with the substance's rewarding effects, such as drug-associated cues. These stimuli can trigger the urge to use drugs (incentive salience) by activating the DA system on their own. The DA levels tend to persist even after the rewarding effects of the drugs have declined (5). Imaging studies of cocaine-addicted individuals have reported higher activity in the PFC during drug expectation than during drug administration (15, 60). This is in agreement with our hypothesis that the expectation value of BQ after 24 h of abstinence in dependent individuals was enhanced and may have contributed to the observed increased FC found in this investigation. Our study found increased FC in the amygdala whose key role is to control stress reactions and negative emotions (82). Therefore, the 24 h abstinence in BQ dependent individuals may have triggered unpleasant feelings associated with withdrawal symptoms which are believed to originate from reduced activation in the reward network of the basal ganglia and increased activation of the stress system including stress neurotransmitters (corticotropin-releasing factor, norepinephrine, and dynorphin) (83) in the amygdala (84). There is evidence that all abused substances tend to disrupt the dopamine reward system when used for a long time (6). For instance, addicts in imaging studies have constantly demonstrated long-term decreased D2 dopamine receptor, compared with non-addicts (85). The overall loss of reward sensitivity may explain compulsive drug use seen in addicts as a way to experience the pleasurable feelings the reward system formerly exerted (86). The desire to get rid of the negative feelings accompanying withdrawal may therefore reinforce continual drug use like the one demonstrated in individuals with BQD. Additionally, compared to HC1 versus HC2, the FC observed in the subcortex between HC1 and BQD1 groups was not significantly increased. This may be due to reduced D2 dopamine receptors (85) in individuals with BQD, which makes them less sensitive to BQ and therefore increases their compulsivity.

We also found increased FC in the right medial SFG, which mirrors a reduced efficiency of response inhibition processes in the PFC (87). The SFG is involved in planning, and motivation, as well as contributing to both stimulation and inhibition of craving. Its activation during responding to smoking cues versus neutral cues is highly correlated with participants' reports of craving (88), suggesting that time spent without BQ may have stimulated craving and thus activated the SFG in the BQD group.

Increased FC was observed in the right angular gyrus and right SPG of BQ dependent individuals. Our results are similar to other cocaine studies that have demonstrated increased FC between frontal–temporal and frontal–parietal brain regions of abstinent chronic cocaine users (89). Similarly, compared to people who have never smoked and former nicotine smokers, current smokers had greater connectivity in the right superior parietal lobe located in the dorsal attention network (90). The dorsal attention network has been linked with attention processing, predominantly in employing top-down control over fundamental sensory operations including visual information and maybe a crucial location for distorting attention (91). Therefore, greater connectivity in this area may indicate an increased propensity to focus one's attention on external signals (90), which may make it harder to abstain from BQ. The angular gyrus plays a crucial role in comprehension, reasoning (92), attention, language memory and self-awareness as well as providing information about self-awareness in the default mode network (DMN) (93). Addicts with dysfunctional DMN may exhibit impairment in disease awareness, need for professional help, and/or drug-seeking behavior (94) which supports what is often depicted in BQD behavior.

The results from this study also demonstrated increased FC in the right ITG. The ITG and SPG are involved in visual and auditory processing (95), and the increased FC in these regions in this study may relate to a perceived improvement in visual and auditory abilities in chronic BQ chewers. Such experiences may facilitate maintenance of BQ chewing, thus leading to dependent behavior.

A number of limitations are noted. First, our study was cross-sectional: we only observed functional connectivity differences in naive and chronic BQ chewers, but cannot infer causality. Future longitudinal neuroimaging BQ studies are crucial to consider the mechanisms fundamental for the neuro-transition from initial BQ use to dependence. Second, the use of other substances, such as cigarettes or alcohol could have influenced the results even though all recruited participants met the inclusion and exclusion criteria. Third, for BQ dependent individuals, we did not take into account the influence of craving on functional connectivity and the duration since last BQ use; which might influence the results. Fourth, for the investigation of the acute effects of chewing betel, it would be useful in future studies to have a control group that did not chew betel but was otherwise scanned similarly.

## Conclusion

This is the first study to examine the acute and chronic effects of BQ concurrently. In controls the effect of acute BQ chewing significantly increased functional connectivity between subcortical regions (including the caudate, putamen, pallidum and thalamus); and visual brain regions (including the bilateral superior occipital gyrus and right middle occipital gyrus networks). These increases may relate to the acutely rewarding and visual effects of betel produced by its arecoline. In habitual users of betel, networks comprising the right medial OFC, right lateral OFC, bilateral inferior temporal gyrus, right angular gyrus, superior parietal gyrus, and right medial superior frontal gyrus had higher functional connectivity as compared to the controls before BQ chewing. These differences may be related to the craving for betel.

## Data Availability Statement

The raw data supporting the conclusions of this article will be made available by the authors, without undue reservation, to any qualified researcher.

## Ethics Statement

The studies involving human participants were reviewed and approved by the Ethics Committee of the Second Xiangya Hospital of Central South University. The patients/participants provided their written informed consent to participate in this study.

## Author Contributions

XH designed the study and collected data. XH and SG analyzed the data. AS, XH, and SG prepared the first draft of the manuscript. AS, XH, SG, ER, and ZL critically revised the content of the manuscript. WP, JZ, HL, and ZX actively participated in writing and revising the manuscript. All authors read and approved the final manuscript.

## Funding

This study was supported by the China Precision Medicine Initiative (2016YFC0906300) and the National Natural Science Foundation of China (Grant nos. 81561168021, 81471362, 81671335, 81701325, 81801353, 11671129, 31671134).

## Conflict of Interest

The authors declare that the research was conducted in the absence of any commercial or financial relationships that could be construed as a potential conflict of interest.

## Abbreviations

BQ, Betel quid; BQD, Betel quid dependence; HC, Healthy controls; FC, Functional connectivity; DA, Dopamine; OFC, Orbitofrontal cortex; CAU, Caudate; THA, Thalamus; PUT, Putamen; SOG, Superior occipital gyrus; MOG, Middle occipital gyrus; SPG, Superior parietal gyrus; SFG, Superior frontal gyrus; ITG, Inferior temporal gyrus; ACC, Anterior cingulate cortex.
