# Supplementary material for: Acute and Chronic Effects of Betel Quid Chewing on Brain Functional Connectivity
Source: Front Psychiatry. 2020 Mar 17;11:198. doi: 10.3389/fpsyt.2020.00198 (PMC7094756; doi:10.3389/fpsyt.2020.00198)
Supplement: Supplementary file 1 [file DataSheet_1.docx]

**Acute and Chronic Effects of Betel Quid Chewing on Brain Functional Connectivity**

Adellah Sariah^1, 2, a^, Shuixia Guo^3, 4, a^, Jing Zuo^5, a^ Weidan Pu^6^, Haihong Liu^7^, Edmund T. Rolls^8, 9^, Zhimin Xue^1^, Zhening Liu^1^, Xiaojun Huang^1,*^

**Supporting Information**

In this study, HC1 and BQD1 are defined as healthy controls and participants with Betel Quid dependence (BQD) respectively, who were scanned before BQ chewing. HC2 and BQD2 are defined as healthy controls and participants with BQD respectively scanned after BQ chewing. In theory, we can compare any pair of subjects. However, in the main text, our interest was on the acute impact and the chronic impact of BQ, so we described the results of HC1 vs HC2 (acute impact) and HC1 vs BQD1 (chronic impact). The results of other comparisons are shown in Table S1 and Table S2, while classification analysis results are shown in Table S3 and Figure S2.

**Table S1:** **Statistical approaches and interpretation. (FDR correction)**

| Comparison: | Statistical analysis |
| --- | --- |
| HC1 vs HC2 | 10 links (THA.R--- PreCG.L, ROL.R-- HES.R, CAU.R--- SOG.L, THA.R ---SOG.R, THA.R-- MOG.R, THA.R ---FFG.L, THA.R ---FFG.R, THA.R --- PoCG.L, IOG.L---MOG.L, PCUN.L--- SMG.L) |
| HC1 vs BQD1 | No difference |
| HC1 vs BQD2 | 1 link (THA.L--- MOG.R) |
| HC2 vs BQD1 | No difference |
| HC2 vs BQD2 | No difference |
| BQD1 vs BQD2 | No difference |

**Table S2:** **Statistical approaches and interpretation. (p<0.001)**

| Comparison: | Statistical analysis |
| --- | --- |
| HC1 vs BQD1 | 6 links |
| HC2 vs BQD2 | 7 links |
| HC1 vs HC2 | 57 links |
| BQD1 vs BQD2 | 8 links |
| HC1 vs BQD2 | 30 links |
| HC2 vs BQD1 | 29 links |

Table S3: Classification results for different groups.

|  | Accuracy/p value | Sensitivity | Specificity | AUC |
| --- | --- | --- | --- | --- |
| HC1 vs BQD2 | 86.54%(<0.001) | 85.71% | 87.5 % | 0.8646 |
| HC1 vs HC2 | 78.57%(<0.001) | 75% | 82.14% | 0.7551 |
| BQD1 vs HC2 | 76.92%(<0.001) | 75% | 78.57% | 0.7842 |
| HC1 vs BQD1 | 73.08 %(<0.001) | 71.45% | 75% | 0.8021 |
| HC2 vs BQD2 | 63.46%(0.02) | 67.86% | 58.33% | 0.6801 |
| BQD1 vs BQD2 | 58.33%(0.03) | 62.5% | 54.17% | 0.5990 |

**Note:** **HC1**-first fMRI scan in healthy controls before betel quid chewing; **HC_2**-second fMRI scan in healthy controls after betel quid chewing; **BQD1**- first fMRI scan in betel quid dependent group before betel quid chewing; **BQD2**-second fMRI scan in betel quid dependent group after betel quid chewing; **AUC**-area under the curve

**Table S4.** **The anatomical regions defined in each hemisphere and their label in the automated anatomical labelling atlas AAL2 (Rolls et al, 2015).** Column 4 provides a set of possible abbreviations for the anatomical descriptions.

| NO. | ANATOMICAL DESCRIPTION | LABEL  aal2.nii.gz | POSSIBLE  ABBREVIATION |
| --- | --- | --- | --- |
| 1,2 | Precentral gyrus | Precentral | PreCG |
| 3, 4 | Superior frontal gyrus, dorsolateral | Frontal_Sup | SFG |
| 5, 6 | Middle frontal gyrus | Frontal_Mid | MFG |
| 7, 8 | Inferior frontal gyrus, opercular part | Frontal_Inf_Oper | IFGoperc |
| 9, 10 | Inferior frontal gyrus, triangular part | Frontal_Inf_Tri | IFGtriang |
| 11, 12 | IFG pars orbitalis, | Frontal_Inf_Orb | IFGorb |
| 13, 14 | Rolandic operculum | Rolandic_Oper | ROL |
| 15, 16 | Supplementary motor area | Supp_Motor_Area | SMA |
| 17, 18 | Olfactory cortex | Olfactory | OLF |
| 19, 20 | Superior frontal gyrus, medial | Frontal_Sup_Med | SFGmedial |
| 21, 22 | Superior frontal gyrus, medial orbital | Frontal_Med_Orb | PFCventmed |
| 23, 24 | Gyrus rectus | Rectus | REC |
| 25, 26 | Medial orbital gyrus | OFCmed | OFCmed |
| 27, 28 | Anterior orbital gyrus | OFCant | OFCant |
| 29, 30 | Posterior orbital gyrus | OFCpost | OFCpost |
| 31, 32 | Lateral orbital gyrus | OFClat | OFClat |
| 33, 34 | Insula | Insula | INS |
| 35, 36 | Anterior cingulate & paracingulate gyri | Cingulate_Ant | ACC |
| 37, 38 | Middle cingulate & paracingulate gyri | Cingulate_Mid | MCC |
| 39, 40 | Posterior cingulate gyrus | Cingulate_Post | PCC |
| 41, 42 | Hippocampus | Hippocampus | HIP |
| 43, 44 | Parahippocampal gyrus | ParaHippocampal | PHG |
| 45, 46 | Amygdala | Amygdala | AMYG |
| 47, 48 | Calcarine fissure and surrounding cortex | Calcarine | CAL |
| 49, 50 | Cuneus | Cuneus | CUN |
| 51, 52 | Lingual gyrus | Lingual | LING |
| 53, 54 | Superior occipital gyrus | Occipital_Sup | SOG |
| 55, 56 | Middle occipital gyrus | Occipital_Mid | MOG |
| 57, 58 | Inferior occipital gyrus | Occipital_Inf | IOG |
| 59, 60 | Fusiform gyrus | Fusiform | FFG |
| 61, 62 | Postcentral gyrus | Postcentral | PoCG |
| 63, 64 | Superior parietal gyrus | Parietal_Sup | SPG |
| 65, 66 | Inferior parietal gyrus, excluding supramarginal and angular gyri | Parietal_Inf | IPG |
| 67, 68 | SupraMarginal gyrus | SupraMarginal | SMG |
| 69, 70 | Angular gyrus | Angular | ANG |
| 71, 72 | Precuneus | Precuneus | PCUN |
| 73, 74 | Paracentral lobule | Paracentral_Lobule | PCL |
| 75, 76 | Caudate nucleus | Caudate | CAU |
| 77, 78 | Lenticular nucleus, Putamen | Putamen | PUT |
| 79, 80 | Lenticular nucleus, Pallidum | Pallidum | PAL |
| 81, 82 | Thalamus | Thalamus | THA |
| 83, 84 | Heschl’s gyrus | Heschl | HES |
| 85, 86 | Superior temporal gyrus | Temporal_Sup | STG |
| 87, 88 | Temporal pole: superior temporal gyrus | Temporal_Pole_Sup | TPOsup |
| 89, 90 | Middle temporal gyrus | Temporal_Mid | MTG |
| 91, 92 | Temporal pole: middle temporal gyrus | Temporal_Pole_Mid | TPOmid |
| 93, 94 | Inferior temporal gyrus | Temporal_Inf | ITG |


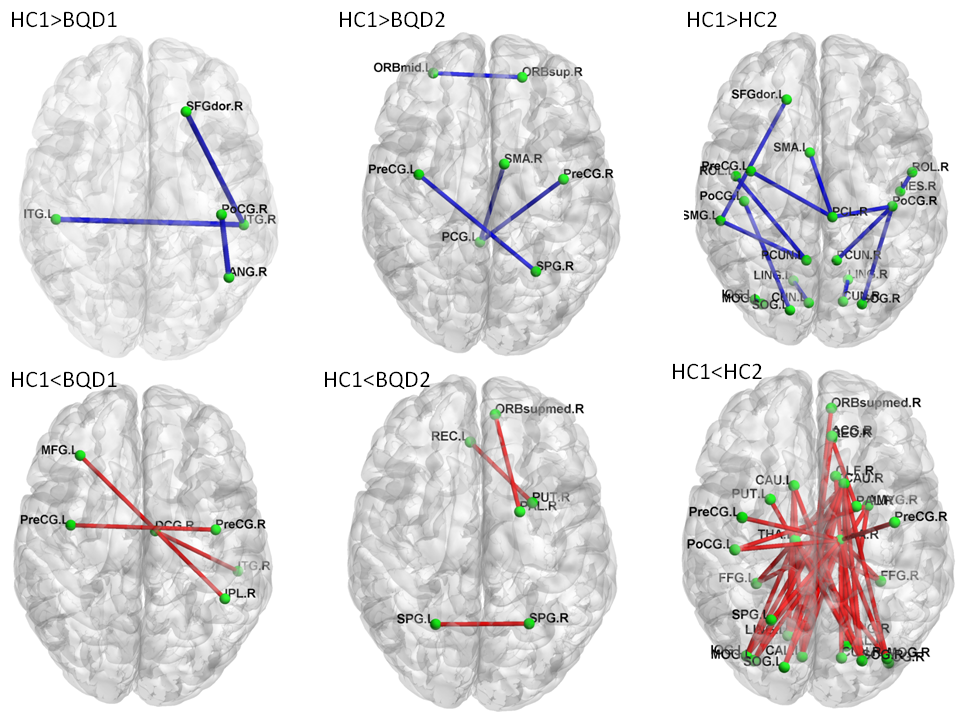


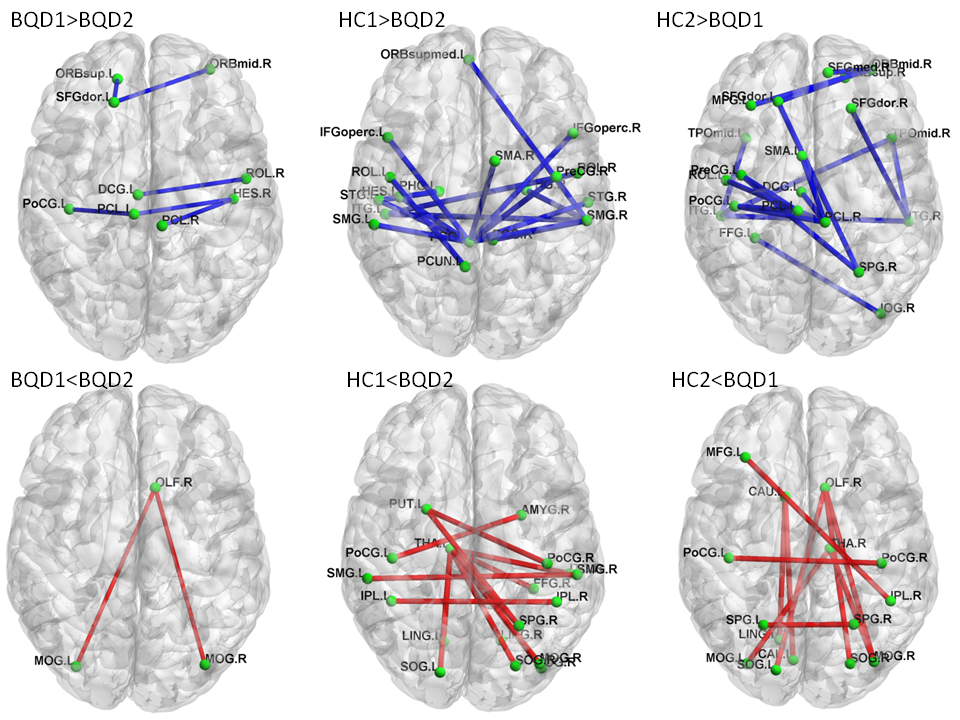


Figure S1: The significant links shown in Table S2.

Figure S2: ROC curve of discrimination between groups. Healthy controls who did not chew betel quid and individuals who chewed betel quid have a greater difference compared to the groups that chewed BQ. HC1, Healthy controls who did not chew betel quid; HC2, healthy controls who chewed betel quid; BQDS1, betel quid dependent chewers who did not chew betel quid; BQDS2, betel quid dependent chewers who chewed betel quid; ROC, Receiver operating characteristics
